# Seizing life with both hands: longitudinal analyses of grip strength among informal caregivers in Europe (SHARE)

**DOI:** 10.1186/s12877-025-05949-y

**Published:** 2025-04-30

**Authors:** Larissa Zwar, Hans-Helmut König, André Hajek

**Affiliations:** 1 Department of Health Economics and Health Services Research, University Medical Center Hamburg-Eppendorf, Hamburg, Germany; 2 Department of Psychology, Brock University, 1812 Sir Isaac Brock Way, St. Catharines, ON L2S 3A1 Canada

**Keywords:** Grip strength, Frailty, Caregiving, Family caregiver, Gender, Old age, Successful aging, Co-residency

## Abstract

**Background:**

This longitudinal study analyzed the association between informal caregiving inside and caregiving outside the household with changes in grip strength, and whether these associations varied based on caregivers’ gender and age among adults in Europe.

**Methods:**

Data from the longitudinal Survey of Health, Ageing and Retirement in Europe (SHARE) was used, including participants aged ≥ 40 years from 10 European countries (pooled over five waves; 2004–2015). Grip strength was measured as maximum grip strength of both hands, informal caregiving as transitions in status as caregiver inside or outside the household. Fixed Effects regression analysis was used, adjusted for health, body mass index and sociodemographic background and additional analyses were conducted with age and gender as moderators.

**Results:**

Higher grip strength was found among those who transitioned into caregiving outside the household. With higher age, the association between caregiving outside the household and grip strength was stronger, and more pronounced among men. The transition into caregiving inside the household was associated with lower grip strength at older age.

**Conclusions:**

The location of caregiving, and caregiver’s age and gender play an important role for changes in grip strength. The findings suggest that caregiving outside the household might be helpful for grip strength, in particular for older and male adults. Older caregivers inside the household, however, seem to need more support to prevent further decline in grip strength.

**Supplementary Information:**

The online version contains supplementary material available at 10.1186/s12877-025-05949-y.

## Background

Among adults aged 50 years and older in Europe, up to 25% provide informal care, i.e. unpaid care for relatives or friends [1]. In research up to date, findings on physical health outcomes are inconclusive [2–5]. The results vary with the health indicator studied, which suggests that caregiving affects only specific aspects of health [4, 6]. One factor that could be of relevance in this context is grip strength.

Grip strength is an objective, easily measured indicator of health status [7] and associated with morbidity [7], frailty [8, 9] and mortality [10]. Findings on the association between caregiving and grip strength would extend our understanding of antecedents of grip strength and would provide an easily measured early warning signal of future health threats among caregivers.

However, grip strength has not received much attention in research on informal caregiving. Two studies analyzed female caregivers aged 65 years and older from a follow-up study before 2000 [11] and between 1999 and 2004 [12] in the US. One found higher grip strength among low-frequency caregivers compared to non-caregivers [11], while the other found best physical functioning (including grip strength) among high-intensity caregivers [12]. A cross-sectional study from Japan found lower grip strength among female but not among male caregivers [13]. Further research indicated that multiple roles throughout life (e.g., work and family roles) are beneficial for grip strength in later life in Europe [14]. These few and inconclusive findings are based mostly on older data from outside Europe and include only two longitudinal data analyses. Further research in Europe with longitudinal data that allows to analyze changes in grip strength in association with the change in caregiver status are thus needed. Moreover, longitudinal research is needed that applies methods that reduce the danger of bias in observational data due to unobserved heterogeneity. Fixed effects (FE) regression have an advantage in this compared to previously used methods (e.g., Mixed Effects, [12]), and will be described in further detail in Statistics [15].

There are also some studies on the physical phenotype of frailty, including grip strength as indicator of weakness [9]. These studies were all from Europe and found mostly higher frailty among caregivers [16–19]. Two studies used data from the Survey of Health, Ageing and Retirement in Europe (SHARE), which will also be used in our study. The cross-sectional study focused only on caregiving inside the household [18], the longitudinal study only on spousal caregiving inside the household [19]. Both found a higher risk of frailty among caregivers. However, the index measure does not provide information on the individual indicators of frailty, such as grip strength. Caregiving is not associated with all indicators of health [4, 6], thus, for a comprehensive understanding of caregiving and its association with health and to develop measures focused on the aspects of health that are most affected, research needs to analyze different health indicators separately. Up to date, the findings provide no clear indication of the change in grip strength among adults transitioning into caregiving. Moreover, previous research focused only on specific subgroups, thus, research is needed that analyzes whether the association depends on specific aspects of the caregiving situation.

### Theoretical frame and hypotheses

According to stress theories, a situation that is perceived as threatening due to insufficiently available resources activates physical and psychological stress responses, which can affect wellbeing and health negatively [20, 21]. Informal caregiving, being a potentially stressful situation, is therefore expected to be associated with a lower grip strength once individuals have begun providing care (i.e. transitioned into caregiving).

However, informal caregiving can be physically demanding, since it involves a wide range of care tasks, from household activities to personal care. Due to this, caregiving could improve grip strength similar to other physical activities [22]. This is in line with the *healthy caregiver hypothesis* [12, 23], that has been supported by previous research. According to this, caregivers may benefit in their physical fitness from caregiving or invest in maintaining their health to be able to continue providing care.

Contextual and individual factors may influence the association between caregiving and grip strength, in line with stress theories [20, 21]. We propose that the context of caregiving, in terms of providing care inside or outside one’s household and individual factors in terms of age and gender of the caregiver are of importance.

Providing care inside the household (CGIH) can differ from caregiving outside the household (CGOH). For example, co-residents are more often spouses and care intensity is higher among co-residing caregivers [24]. Also, co-residing caregivers, i.e. CGIH, care for individuals with more limitations in activities of daily living [25]. Adding to this, CGIH are often older and include more men than CGOH [25, 26].

Previous findings indicated that CGIH is associated with worse health than CGOH and non-caregiving [27]. Moreover, higher age increases the risk for higher caregiver burden [28] and female compared to male gender is associated with poorer mental health [2]. Interactions of age and gender regarding associations of caregiving and health have been found as well [29]. Adding to this, strong evidence points to gender and age differences in grip strength [30]. On average, women have lower levels of grip strength (around 20 kg) than men [30]. Grip strength is also associated with age, peaking at around 40 years, and declining thereafter, more so in men than in women [30, 31].

Thus, both CGIH and CGOH are two different forms of care that need to be analyzed separately in their association with health outcomes and regarding gender and age differences. Based on previous findings, we expect different associations between caregiving and grip strength among CGIH and CGOH. Also, we expect a larger change of grip strength among men and with increasing age– either in terms of worsening or improving grip strength. This study will be the first to explore these associations with a large European sample of female and male adults aged 40 years and older, using a longitudinal study design.

## Methods

### Design, setting and sample

Data from the longitudinal Survey of Health, Ageing and Retirement in Europe (SHARE, release 8.0.0) [32, 33] was used, which collects data from people aged 50 + years and their partners (regardless of age), living in the same household with probability sampling [32, 34, 35]. We included all participants from the age of 40 years, since caregivers are primarily found in this age group [26], thus, including also adults who had been sampled as partners. The data included was from waves 1, 2, 4, 5 and 6 (2004 to 2015). Wave 3, 7 and 8 were excluded due to differences in the survey and data unavailability. We used data from participants from Austria, Germany, Sweden, Netherlands, Spain, Italy, France, Denmark, Switzerland, and Belgium, who had participated in all included waves. This resulted in 171,848 observations (see Fig. 1, Supplementary Material). We analyzed changes over time, therefore only those individuals who experienced changes in the analyzed variables were used for the estimation of the regression coefficients. For example, 4,165 transitions were found into caregiving inside the household (more details in Statistics). The first four waves were approved by the Ethics Committee of the University of Mannheim and the continuation of the project by the Ethics Council of the Max Planck Society (detailed information at https://share-eric.eu/data/faqs-support); participants gave informed consent.

### Variables

**Outcome**. Grip strength was measured with a handheld dynamometer (Smedley, S Dynamometer, TTM, Tokyo, 100 kg) by a trained interviewer. Participants stood or sat with their elbow at ninety degrees and their upper arm tight against their trunk with the dynamometer adjusted to their hand and squeezed as hard as possible [36]. The values range between 1 and 99 kg. All participants with values above or below this range were excluded. Valid measures included at least two measures with one hand or two measures for both hands. In total, 8.15% had no valid measures in the pooled sample, which was the basis of our analysis. A maximum grip value was calculated, which provides the maximum measurement based on either all measurements of both hands or of both measurements of one hand. While one grip strength measurement may not provide sufficient information, analyzing changes in grip strength over time and in association with major events, such as caregiving, can provide a useful indicator of health and longevity [7–9].

**Main predictors.** Informal caregiving was defined as providing help for a family member, a friend, or a neighbor outside the household within the last twelve months (no, yes). Help could include any combination of personal care (e.g., dressing, bathing or showering, eating, getting in or out of bed, using the toilet), practical household help (e.g., with home repairs, gardening, transportation, shopping, household chores), or help with paperwork (e.g., filling out forms, settling financial or legal matters), and could occur in frequency from daily to less than monthly. Informal caregiving inside the household was measured in a different way, by asking whether a person provided help on a regular basis (daily or almost daily) for someone in the same household with personal care (e.g., washing, dressing; no, yes) during the last twelve months. Only participants with more than one person in their household were asked this.

**Covariates**. Measured sociodemographic variables included gender, age, education, marital and employment status. Additional health variables, which were used as covariates, included body mass index (based on height and weight), self-rated health (single item of the SF-36 questionnaire [37], Range: 1 excellent to 5 poor health) and the number of chronic diseases (sum score of a list of self-reported chronic diseases, which had been diagnosed by a physician including: heart attack, high blood pressure or hypertension, high blood cholesterol, stroke, diabetes or high blood sugar, chronic lung disease, asthma, arthritis, osteoporosis, cancer, stomach or duodenal ulcer, peptic ulcer, Parkinson disease, cataracts, and hip fracture or femoral fracture, Range: 0–14). Also, variables measuring the engagement in activities requiring a moderate level of energy and sports or activities that are vigorous were measured (rated on 4-point scale from hardly ever/never to more than once a week).

### Statistical analysis

Linear FE regression analyses were conducted [15]. Longitudinal data has the advantage of being able to differentiate between time-constant and time-varying errors. FE regression analysis uses this advantage by controlling all time-constant observed and unobserved variables. Due to this, time-constant unobserved variables which may be associated with the analyzed variables cannot bias the estimates, such as genetic disposition [38]. Thus, if time-varying heterogeneity is controlled, consistent parameters can be estimated with this method. Therefore, this method is ideally suited to test whether two variables are associated, while reducing the danger of bias significantly. In line with theoretical considerations and previous findings [14, 39], all models were adjusted for health and sociodemographic factors. Gender and education were already controlled due to them being time-constant variables and therefore not added. Moderator analyses were conducted with gender and age as moderators. To account for clusters within the data due to inclusion of different countries and multiple waves, we calculated cluster-robust standard errors [40]. Additional analyses were conducted to test the robustness of the findings (see Supplementary Material). The main analyses for both CGIH and CGOH were stratified by age groups (40 to 59, 60 to 79, 80 and older; Table A2). Asymmetric FE regression analysis was used to test transitioning into (beginning) and out of (ending) caregiving in addition (Table A3 and A4). For CGOH we repeated the analysis with frequency of CGOH (measured as 1 daily, 2 weekly, 3 monthly, 4 less than monthly for at least one person with care needs) as main predictor (Table A5).

The majority of the variables had low levels of missing values (Table A1, Supplementary Material), thus, listwise deletion was used in line with literature recommendations [41, 42]. Moreover, FE regression analyses further reduces the danger of bias due to selection, panel attrition and missing values, since it controls for all (observed and unobserved) time-constant variables [15, 43]. The level of significance was set at α = 0.05 and analyses were conducted with Stata 16.1 (Stata Corp., College Station Texas).

## Results

### Descriptive results

The selection process is illustrated in Fig. 1 (Supplemental file). In Table 1 the complete sample pooled over all five waves and the two groups providing care inside (7.47% of those not living alone) and outside the household (26.18%) are described. The CGOH were on average 62.89 (SD = ± 8.83) years and 56.48% were female. They provided care for parents, stepparents or parents-in-laws (32.79%), other relatives (33.76%) or non-relatives (30.52%); only 2.87% provided care for their partner. CGIH were older (M = 67.75, SD = ± 10.85) and included more women (58.28%). The majority provided care for their partner (64.46%), their parents (14.81%), or other relatives (11.13%). The data contained 9,667 transitions into caregiving outside the household. Among all who were not living alone, 4,165 transitions into caregiving inside the household were found. The maximum grip strength among CGIH was 32.01 kg (SD = ± 11.80) and 36.23 kg (SD = ± 11.75) among CGOH.

**Table 1:** Description of the analytical sample(s) pooled over waves 1, 2, 4, 5 and 6 (2004 to 2015) of the SHARE data

*N*(%)	Complete Sample (*N* = 171,848)			
Caregiving	…inside the household^1^		…outside the household	
- Yes	10,148 (7.47)		44,998 (26.18)	
- No	124,505 (91.62)		99,915 (58.14)	
- Transitions into… (Frequencies)	4,165		9,667	
Who is cared for…^2^				
- Partner/spouse	6,541 (64.46)		1,290 (2.87)	
- Parents/ Stepparents/ Parents-in-law	1,503 (14.81)		14,754 (32.79)	
- Other relatives	1,129 (11.13)		15,193 (33.76)	
- Non-relatives	154 (1.52)		13,732 (30.52)	
***N*** **(%)/M(SD)**	**Complete sample**	**Caregivers inside the household** ^**1**^		**Caregivers outside the household**
Age	66.27 (10.47) [Range: 40–105]	67.75 (10.85) [Range: 40–100]		62.89 (8.83) [Range: 40–103]
Age groups				
- 40 to 59	54,682 (31.82)	2,837 (27.96)		19,026 (42.28)
- 60 to 70	96,911 (56.39)	5,750 (56.66)		24,058 (53.46)
- 80 and older	20,255 (11.79)	1,561 (15.38)		1,914 (4.25)
Gender				
- Male	76,842 (44.72)	4,234 (41.72)		19,584 (43.52)
- Female	95,006 (55.28)	5,914 (58.28)		25,414 (56.48)
Education (ISCED 1997)				
- None/ still in school /other	9,660 (5.62)	868 (8.55)		1,040 (2.31)
- Primary education (Code 1)	35,772 (20.82)	2,659 (26.20)		5,895 (13.10)
- Lower secondary education (Code 2)	29,156 (16.97)	1,798 (17.72)		7,221 (16.05)
- Upper secondary education (Code 3)	51,519 (29.98)	2,707 (26.68)		15,338 (34.09)
- Post-secondary non-tertiary education (Code 4)	5,294 (3.08)	233 (2.30)		1,712 (3.80)
- First stage of tertiary education (Code 5)	36,691 (21.35)	1,663 (16.39)		13,038 (28.97)
- Second stage of tertiary education (Code 6)	1,437 (0.84)	71 (0.70)		419 (0.93)
BMI	26.42 (4.49)	26.80 (4.65)		26.20 (4.36)
Marital status				
- Married and living together with spouse/ partner	118,614 (69.02)	8,489 (83.65)		30,329 (67.40)
- Registered partnership	2,876 (1.67)	174 (1.71)		893 (1.98)
- Married, living separated from spouse/ partner	1,935 (1.13)	68 (0.67)		625 (1.39)
- Never married	10,133 (5.90)	434 (4.28)		3,033 (6.74)
- Divorced	13,603 (7.92)	291 (2.87)		5,096 (11.32)
- Widowed	23,475 (13.66)	626 (6.17)		4,854 (10.79)
Current employment status				
- Retired	89,286 (51.96)	5,569 (54.88)		20,327 (45.17)
- Employed or self-employed	48,103 (27.99)	2,041 (20.11)		16,899 (37.56)
- Unemployed	4,892 (2.85)	271 (2.67)		1,554 (3.45)
- Permanently sick or disabled	5,679 (3.30)	448 (4.41)		1,412 (3.14)
- Homemaker	19,225 (11.19)	1,636 (16.12)		4,063 (9.03)
- Other	2,275 (1.32)	157 (1.55)		609 (1.35)
Self-rated health	3.02 (1.07)	3.31 (1.08)		2.76 (1.03)
Number of chronic diseases	1.08 (1.18)	1.28 (1.28)		0.92 (1.07)
Activities requiring a moderate level of energy	3.40 (1.04)	3.23 (1.18)		3.66 (0.76)
Sports or activities that are vigorous	2.39 (1.35)	10,143 (2.23)		2.73 (1.31)
Hand grip strength	34.04 (11.97)	32.01 (11.80)		36.23 (11.75)

Note. Pooled dataset based on data from waves 1, 2, 4, 5 and 6 (2004 to 2015) from the following countries were included: Austria, Germany, Sweden, Netherlands, Spain, Italy, France, Denmark, Switzerland, and Belgium

^1^ only those who were not living alone were asked if they were providing care inside the house; 10.28% of those having missing information on caregiving were living alone and therefore not asked if they provided care for anyone inside their household

^2^ for caregivers outside the house, information on who is cared for is only provided for the care recipient mentioned first (main care recipient)

### Results of regression analyses

Transitioning into CGOH was associated with increased grip strength (b = 0.19, CI[0.11; 0.27], *p* < 0.001) in adjusted FE regression analyses (Table 2, model 1). Moderator analyses (Table 2) indicated statistically significant interaction effects between CGOH and age (b = 0.02, CI[0.01; 0.03], *p* < 0.001, model 2), and gender (b=-0.34, CI[-0.51; -0.18], *p* < 0.001, model 3). The association between transitioning into CGOH and increased grip strength was stronger with higher age and stronger among men compared to women. The three-way interaction between transitioning into CGOH, age, and gender was statistically significant (b=-0.03, CI[-0.05; -0.01], *p* < 0.01, Table 2, model 4). With increasing age, transitioning into CGOH was associated with a larger grip strength, which was significantly stronger among men.

**Table 2:** Fixed effects regression analysis with the complete sample (model 1), moderator analysis with age (model 2) and gender (model 3) and three-way interaction between caregiving, gender and age (mode 4) for the main predictor *caregiving outside the household*

	(1)			(2)			(3)			(4)		
	Main analysis			Moderator age			Moderator gender			Moderators age and gender		
VARIABLES	b	Robust SE	CI	b	Robust SE	CI	b	Robust SE	CI	b	Robust SE	CI
Caregiving outside the household (ref. No)	0.19***	(0.04)	0.11–0.27	-1.20***	(0.29)	-1.77 - -0.64	0.38***	(0.07)	0.25–0.52	-2.03***	(0.51)	-3.03 - -1.04
Age at interview (in years)	-0.39***	(0.01)	-0.40 - -0.38	-0.39***	(0.01)	-0.41 - -0.38	-0.39***	(0.01)	-0.40 - -0.38	-0.53***	(0.01)	-0.55 - -0.51
Caregiving outside the household (ref. No) x Age at interview (in years)				0.02***	(0.00)	0.01–0.03				0.04***	(0.01)	0.02–0.05
Gender (ref. male)							-		-	-		-
Caregiving outside the household (ref. No) x Gender (ref. Male)							-0.34***	(0.08)	-0.51 - -0.18	1.73**	(0.60)	0.55–2.90
Gender (ref. Male) x Age at interview (in years)										0.25***	(0.01)	0.23–0.27
Caregiving outside the household (ref. No) x Gender (ref. Male) x Age at interview (in years)										-0.03**	(0.01)	-0.05 - -0.01
Constant	56.03***	(0.53)	54.99–57.07	56.50***	(0.55)	55.43–57.57	56.05***	(0.53)	55.00–57.09	56.71***	(0.54)	55.64–57.77
Observations	129,891			129,891			129,891			129,891		
N	63,828			63,828			63,828			63,828		
R²	0.12			0.12			0.12			0.13		

Note. Unstandardized regression coefficients of Fixed Effects regression analysis with robust standard errors in parentheses. All models are adjusted for age, marital status, current employment status, body mass index, activities requiring a moderate level of energy and sports or activities that are vigorous, and self-perceived health and number of chronic diseases. Level of significance: *** *p* < 0.001, ** *p* < 0.01, * *p* < 0.05, + *p* < 0.10

Transitioning into CGIH was not associated with changes in grip strength (b=-0.01, CI[-0.16, 0.14], Table 3, model 1). Gender did not moderate this (model 3), however, a statistically significant interaction effect between CGIH and age was found (b=-0.02, CI[-0.04; -0.01], *p* < 0.01, model 2). With increasing age, transitioning into CGIH was associated with lower grip strength. The three-way interaction was not significant (model 4).

**Table 3:** Fixed effects regression analysis with the complete sample (model 1), moderator analysis with age (model 2) and gender (model 3) and three-way interaction between caregiving, gender and age (mode 4) for the main predictor *caregiving inside the household*

	(1)			(2)			(3)			(4)		
	Main analysis			Moderator age			Moderator gender			Moderators age and gender		
VARIABLES	b	Robust SE	CI	b	Robust SE	CI	b	Robust SE	CI	b	Robust SE	CI
Caregiving inside the household (ref. No)	-0.01	(0.08)	-0.16–0.14	1.48**	(0.50)	0.50–2.47	-0.08	(0.13)	-0.35–0.18	2.49**	(0.88)	0.77–4.22
Age at interview (in years)	-0.39***	(0.01)	-0.40 - -0.38	-0.39***	(0.01)	-0.40 - -0.37	-0.39***	(0.01)	-0.40 - -0.38	-0.50***	(0.01)	-0.52 - -0.48
Caregiving inside the household (ref. No) x Age at interview (in years)				-0.02**	(0.01)	-0.04 - -0.01				-0.04**	(0.01)	-0.06 - -0.01
Gender (ref. male)							-		-	-		-
Caregiving inside the household (ref. No) x Gender (ref. male)							0.13	(0.16)	-0.19–0.45	-1.60	(1.06)	-3.68–0.47
Gender (ref. male) x Age at interview (in years)										0.23***	(0.01)	0.21–0.25
Caregiving inside the household (ref. No) x Gender (ref. Male) x Age at interview (in years)										0.02	(0.02)	-0.01–0.05
Constant	56.27***	(0.57)	55.16–57.39	56.15***	(0.57)	55.03–57.27	56.27***	(0.57)	55.15–57.39	56.37***	(0.57)	55.26–57.49
Observations	121,804			121,804			121,804			121,804		
N	56,760			56,760			56,760			56,760		
R²	0.10			0.10			0.10			0.11		

Note. Unstandardized regression coefficients of Fixed Effects regression analysis with robust standard errors in parentheses. All models are adjusted for age, marital status, current employment status, body mass index, activities requiring a moderate level of energy and sports or activities that are vigorous, and self-perceived health and number of chronic diseases. Level of significance: *** *p* < 0.001, ** *p* < 0.01, * *p* < 0.05, + *p* < 0.10

After stratifying the main analysis by different age groups (Table A2), the significant association between caregiving and grip strength was only found for age group 1 (40 to 59; b = 0.20, *p* < 05) and age group 2 (60 to 79; b = 0.16, *p* < 0.01).

Additional asymmetric FE (Table A3) for CGOH indicated beginning (b = 0.34, *p* < 0.001) and ending CGOH were associated with higher grip strength (b = 0.13, *p* < 0.05). The three-way interaction was significant (b = 0.04, *p* < 0.01) only for ending caregiving, with women showing more grip strength with increasing age than men in association with ending care. Among CGIH (Table A4) neither beginning (b=-0.05, *p* = 0.634) nor ending CGIH (b=-0.17, *p* = 0.155) was significantly associated with grip strength. Three-way interaction for both beginning (b = 0.06, *p* < 0.01) and ending CGIH (b = 0.05, *p* < 0.05) were both significant, indicating women to have higher grip strength with older age in association with ending care. Last, frequency of CGOH was not significantly associated with grip strength and did not interact with either age or gender (Table A5).

## Discussion

This study analyzed whether transitions in two different forms of caregiving (inside or outside the household) were associated with changes in grip strength and what role the caregiver’s age and gender played. Our findings highlight the importance of distinguishing between GCIH and CGOH when exploring indicators of physical health. They substantially add to the sparse and inconclusive results that have addressed grip strength among informal caregivers previously and are the first to employ an FE approach.

CGIH was not related to changes in caregivers’ grip strength. CGIH comprised mainly partner care. A different level of burden and expectation to provide care is associated with selecting into this form of care, further supporting the entanglement of the location of care and care relationship and their connection with health indicators among caregivers [24, 25, 44, 45].

However, CGOH was related to grip strength, with a transition into this form of caregiving being associated with higher levels of grip strength. The magnitude of the changes was small, but nonetheless significant. Additional analyses confirmed higher grip strength associated with beginning and ending caregiving. This partially supports the healthy caregiver hypothesis [12, 23] and aligns with the differences characterizing CGOH compared to CGIH. CGOH were younger, healthier, fewer were married, and more were self-employed than CGIH, and they mostly cared for relatives and non-relatives, but only few for a partner. Thus, individuals who were more fit and flexible in their time-management may have selected into this caregiving and caregiving performance itself may have a strengthening effect on this group. This adds to previous findings on positive effects of caregiving [46] and to findings from the US with older data sets, which focused only on older, female caregivers [11, 12]. However, considering ending care was also associated with a significant higher level of grip strength, this could also hint at a recovering effect after care provision. Future longitudinal analysis tracking changes over the caregiving period from beginning to end could provide further clues about the development of grip strength throughout the caregiving period.

Further findings indicate that the associations partially vary with age and gender. Among CGOH the association between caregiving and grip strength was stronger with higher age and among men. CGOH may mitigate the usual decline of grip strength in the second half of life [30] among older caregivers and thus reduce the risk to health and longevity for which it is a predictive factor [7, 27, 47]. However, this may not be the case for those aged 80 years and older, according to additional analyses. Adding to this, asymmetric analyses showed only a significant interaction between age, gender and CGOH among those ending care. With increasing old age, ending care was associated with higher grip strength among women ending care, further highlighting a possible recovery effect found in this group. Based on the correlational nature of the analysis, selecting into CGOH may also be the result of a slower decline of grip strength and older women with stronger grip strength may be more inclined to end caregiving.

Adding to this, older male CGOH had the strongest grip strength. On average, men have greater grip strength than women, but it decreases more strongly with age [30]. Our findings suggest that (older) men experience this association between CGOH and grip strength more than (younger) female caregivers. Since men usually have lower life expectancy than women [48], CGOH may be helpful to mitigate negative effects of aging, supporting their health and life expectancy. It is also possible that older men with higher levels of grip strength are more likely to select into CGOH, while women provide care irrespective of this physical health indicator. The magnitude of the association was small and additional asymmetric effects could not detect a significant interaction for beginning with care. Further research on the underlying mechanisms of this gender difference is thus recommended.

Age also moderated the non-significant association between caregiving and grip strength. With increasing age, beginning and end of CGIH was associated with reduced grip strength. This extends our initial findings and may be explained by changing intensity of caregiving, although previous findings on the relevance of caregiving intensity are inconclusive [11, 12]. However, our data does not entail information on care intensity inside the household, thus, future research is recommended to analyze this in further detail. While stratifying by age group revealed no further insights, asymmetric analysis found an interaction between age, CGIH and gender. With increasing age, women beginning or ending CGIH had stronger grip strength than men. Once again this highlights the different characters of CGIH and CGOH.

Last, our findings further highlight that frailty and grip strength should be analyzed separately. Findings on frailty all indicated a detrimental effect of informal caregiving [16–19]. However, frailty is an index based on various indicators of physical functioning and different forms of caregiving seem to be associated with these indicators in a different way, as has also been indicated by previous findings [4, 6, 16].

Limiting our study’s findings is the possibility of a selection bias due to panel attrition and non-response [49]. However, previous analyses on bias by demographic or health variables have shown these biases to be either very small or non-existent [32]. Reverse causality is possible as well as bias due to unobserved time-variant heterogeneity. However, random or mixed effects models as well as structural equation models are also faced by these dangers in addition to the bias due to unobserved time-constant heterogeneity [15, 43]. In contrast to these, FE regression has the advantage of reducing the danger of bias due to time-constant variables by controlling for all observed and unobserved time-constant variables [15, 43]. This is of great advantage when working with observational data [50]. Additional research employing methods, such as instrumental variable estimation could add to our research in testing causal associations but this relies heavily on carefully choosing appropriate instrumental variables [50].

Adding to this, the gap between the waves was 2 to 4 years. Caregivers could have transitioned into caregiving at the beginning or end of this period, which could result in different experiences. The danger of this possible bias is larger for those transitioning between the two waves that are 4 years apart and they may be affected differently, due to, for example, having been caregivers for a longer period at the subsequent wave than other participants. It is a danger that all longitudinal designs collecting data with large gaps between assessment times are faced with, since the danger of this bias needs to be weighed against the burden for participants due to more frequent questioning.

Findings refer to the population of adults in middle age (40 years and older) in Europe. Younger caregivers may have different experiences, since grip strength changes with age, and is primarily increasing up to the age of 40 [30, 31]. Thus, further analyses with younger caregiver cohorts are needed to provide more detailed information on the generalizability of our results on these groups.

## Conclusion

In summary, the findings add new evidence with a large population-based sample from Europe. They indicate that caregiving outside the household is associated with better grip strength, and this effect was stronger with increasing age, especially among male caregivers. This may add to other positive effects of caregiving such as providing a new purpose in life [46, 51]. Outside-the-household caregiving could thus contribute to better health and successful aging [52], especially among older and male adults.

In contrast, caregiving inside the household was associated with worsening grip strength with increasing age. Thus, caregivers inside the household may benefit from more support to prevent a worsening of grip strength and associated health outcomes [7].

Changes in grip strength could be used as an easily measured early warning signal of burden [53]. This is mainly important among older CGIH, according to our results. Caregivers inside the household, primarily spouses, are often hidden and do not ask for help [54, 55], although they provide more intensive care [44], have less opportunities for respite, and more negative health effects of caregiving than other caregivers [27]. Grip strength measurements could be used, for example by general practitioners in regular check-ups, as an indicator and opportunity to enquire about caregiving responsibilities and needs in one’s household to be able to identify and effectively support caregivers inside the household.

## Electronic supplementary material

Below is the link to the electronic supplementary material.

Supplementary Material 1

## Data Availability

Data from of the Survey of Health, Ageing and Retirement in Europe (SHARE), release 8.0.0 (10th Feb. 2022) of waves 1 (DOI: 10.6103/SHARE.w1.800), 2 (DOI: 10.6103/SHARE.w2.800), 4 (DOI: 10.6103/SHARE.w4.800), 5 (DOI:10.6103/SHARE.w5.800) and 6 (DOI: 10.6103/SHARE.w6.800) and SHAREeasy (10.6103/SHARE.easy.800) was used, see Börsch-Supan et al. (2013) and Gruber et al. (2014) for methodological details. Data is available free of charge for registered users at http://www.share-project.org. The study was not preregistered.

## Abbreviations

SHARE: Survey of Health, Ageing and Retirement in Europe
CGIH: Caregiving inside the household
CGOH: Caregiving outside the household
FE: Fixed effects

## References

[1] Tur-Sinai A, Teti A, Rommel A, Hlebec V, Lamura G. How many older informal caregivers are there in Europe? Comparison of estimates of their prevalence from three European surveys. Int J Environ Res Public Health. 2020;17(24):9531. 33352669 10.3390/ijerph17249531 PMC7767284

[2] Bom J, Bakx P, Schut F, van Doorslaer E. The impact of informal caregiving for older adults on the health of various types of caregivers: A systematic review. Gerontologist. 2019;59(5):e629–42. 30395200 10.1093/geront/gny137 PMC6850889

[3] Janson P, Willeke K, Zaibert L, Budnick A, Berghofer A, Kittel-Schneider S, et al. Mortality, morbidity and Health-Related outcomes in informal caregivers compared to Non-Caregivers: A systematic review. Int J Environ Res Public Health. 2022;19(10):5864. 35627399 10.3390/ijerph19105864 PMC9141545

[4] Kaschowitz J, Lazarevic P. [Importance of health indicators in the analysis of health effects on informal caregivers: different indicator, different result?]. Z Gerontol Geriatr. 2020;53(1):10–6. 31802211 10.1007/s00391-019-01663-8

[5] Allen AP, Curran EA, Duggan A, Cryan JF, Chorcorain AN, Dinan TG, et al. A systematic review of the Psychobiological burden of informal caregiving for patients with dementia: focus on cognitive and biological markers of chronic stress. Neurosci Biobehav Rev. 2017;73:123–64. 27986469 10.1016/j.neubiorev.2016.12.006

[6] Zwar L, Konig HH, Hajek A. Consequences of different types of informal caregiving for mental, self-rated, and physical health: longitudinal findings from the German ageing survey. Qual Life Research: Int J Qual Life Aspects Treat Care Rehabilitation. 2018;27(10):2667–79. 10.1007/s11136-018-1926-0 29956109

[7] Bohannon RW. Grip strength: an indispensable biomarker for older adults. Clin Interv Aging. 2019;14:1681–91. 31631989 10.2147/CIA.S194543 PMC6778477

[8] Sousa-Santos AR, Amaral TF. Differences in handgrip strength protocols to identify sarcopenia and frailty - a systematic review. BMC Geriatr. 2017;17(1):238. 29037155 10.1186/s12877-017-0625-y PMC5644254

[9] Fried LP, Tangen CM, Walston J, Newman AB, Hirsch C, Gottdiener J, et al. Frailty in older adults: evidence for a phenotype. J Gerontol Biol Sci Med Sci. 2001;56(3):M146–56. 10.1093/gerona/56.3.m146 11253156

[10] Cai Y, Liu L, Wang J, Gao Y, Guo Z, Ping Z. Linear association between grip strength and all-cause mortality among the elderly: results from the SHARE study. Aging Clin Exp Res. 2021;33(4):933–41. 32524391 10.1007/s40520-020-01614-z

[11] Rosso AL, Lee BK, Stefanick ML, Kroenke CH, Coker LH, Woods NF, et al. Caregiving frequency and physical function: the women’s health initiative. J Gerontol Biol Sci Med Sci. 2015;70(2):210–5. 10.1093/gerona/glu104 PMC4311185 25060315

[12] Fredman L, Doros G, Ensrud KE, Hochberg MC, Cauley JA. Caregiving intensity and change in physical functioning over a 2-year period: results of the caregiver-study of osteoporotic fractures. Am J Epidemiol. 2009;170(2):203–10. 19443666 10.1093/aje/kwp102 PMC2727270

[13] Hori Y, Hoshino J, Suzuki K. Physical and psychological health problems among Japanese family caregivers. Nagoya J Med Sci. 2011;73(3–4):107–15. 21928692 PMC4831219

[14] Uccheddu D, Emery T, Gauthier AH, Steverink N. Gendered work-family life courses and late-life physical functioning: A comparative analysis from 28 European countries. Adv Life Course Res. 2022;53:100495. 36652213 10.1016/j.alcr.2022.100495

[15] Wooldridge JM. Econometric analysis of cross section and panel data. MIT Press; 2010.

[16] Potier F, Degryse JM, Bihin B, Debacq-Chainiaux F, Charlet-Renard C, Martens H, et al. Health and frailty among older spousal caregivers: an observational cohort study in Belgium. BMC Geriatr. 2018;18(1):291. 30477431 10.1186/s12877-018-0980-3 PMC6258488

[17] Potier F, Degryse JM, Aubouy G, Henrard S, Bihin B, Debacq-Chainiaux F, et al. Spousal caregiving is associated with an increased risk of frailty: A Case-Control study. J Frailty Aging. 2018;7(3):170–5. 30095147 10.14283/jfa.2018.11

[18] Barbosa F, Voss G, Delerue Matos A. Do European co-residential caregivers aged 50 + have an increased risk of frailty? Health Soc Care Community. 2020;28(6):2418–30. 32557977 10.1111/hsc.13064 PMC7818189

[19] Uccheddu D, Gauthier AH, Steverink N, Emery T. The pains and reliefs of the transitions into and out of spousal caregiving. A cross-national comparison of the health consequences of caregiving by gender. Soc Sci Med. 2019;240:112517. 31561110 10.1016/j.socscimed.2019.112517

[20] Lazarus RS, Folkman S. Stress, appraisal, and coping. Springer publishing company; 1984.

[21] Pearlin LI, Mullan JT, Semple SJ, Skaff MM. Caregiving and the stress process: an overview of concepts and their measures. Gerontologist. 1990;30(5):583–94. 2276631 10.1093/geront/30.5.583

[22] Bao W, Sun Y, Zhang T, Zou L, Wu X, Wang D, et al. Exercise programs for muscle mass, muscle strength and physical performance in older adults with sarcopenia: A systematic review and Meta-Analysis. Aging Dis. 2020;11(4):863–73. 32765951 10.14336/AD.2019.1012 PMC7390512

[23] Li L, Wister AV, Mitchell BA. Examination of the healthy caregiver effect among older adults: findings from the Canadian longitudinal study on aging. Gerontology. 2023;69(3):289–300. 36167035 10.1159/000526251

[24] Zhang Y, Bennett MR. Insights Into Informal Caregivers’ Well-being: A Longitudinal Analysis of Care Intensity, Care Location, and Care Relationship. J Gerontol B Psychol Sci Soc Sci. 2024;79(2). 10.1093/geronb/gbad166 PMC10832593 38299971

[25] Lee K, Tang W, Cassidy J, Seo CH, Zhao J, Horowitz A. The impact of formal and informal support on emotional stress among non-co-resident caregivers of persons with dementia. Aging Ment Health. 2022;26(8):1604–12. 34114901 10.1080/13607863.2021.1935460

[26] Rothgang H, Müller R. BARMER Pflegereport 2018. Schriftenreihe Zur gesundheitsanalyse. editor. Siegburg: Asgard;: BARMER; 2018.

[27] Kaschowitz J, Brandt M. Health effects of informal caregiving across Europe: A longitudinal approach. Soc Sci Med. 2017;173:72–80. 27930918 10.1016/j.socscimed.2016.11.036

[28] Tsai CF, Hwang WS, Lee JJ, Wang WF, Huang LC, Huang LK, et al. Predictors of caregiver burden in aged caregivers of demented older patients. BMC Geriatr. 2021;21(1):59. 33446114 10.1186/s12877-021-02007-1 PMC7809883

[29] Marks NF, Lambert JD, Choi H. Transitions to caregiving, gender, and psychological Well-Being: A prospective U.S. National study. J Marriage Family. 2004;64(3):657–67.

[30] Dodds RM, Syddall HE, Cooper R, Kuh D, Cooper C, Sayer AA. Global variation in grip strength: a systematic review and meta-analysis of normative data. Age Ageing. 2016;45(2):209–16. 26790455 10.1093/ageing/afv192 PMC4776623

[31] Massy-Westropp NM, Gill TK, Taylor AW, Bohannon RW, Hill CL. Hand grip strength: age and gender stratified normative data in a population-based study. BMC Res Notes. 2011;4(1):127. 21492469 10.1186/1756-0500-4-127 PMC3101655

[32] Börsch-Supan A, Brandt M, Hunkler C, Kneip T, Korbmacher J, Malter F, et al. Data resource profile: the survey of health, ageing and retirement in Europe (SHARE). Int J Epidemiol. 2013;42(4):992–1001. 23778574 10.1093/ije/dyt088 PMC3780997

[33] Gruber S, Hunkler C, Stuck S. Generating EasySHARE: guidelines, structure, content and programming. SHARE Work Pap Ser. 2014.

[34] Malter F, Börsch-Supan AE. SHARE wave 5: innovations & methodology. Munich: MEA, Max Planck Institute for Social Law and Social Policy; 2015.

[35] Malter F, Börsch-Supan AE. SHARE wave 6: panel innovations and collecting dried blood spots. Munich: MEA, Max Planck Institute for Social Law and Social Policy.; 2017.

[36] Andersen-Ranberg K, Petersen I, Frederiksen H, Mackenbach JP, Christensen K. Cross-national differences in grip strength among 50 + year-old Europeans: results from the SHARE study. Eur J Ageing. 2009;6(3):227–36. 28798606 10.1007/s10433-009-0128-6 PMC5547372

[37] Ware JE Jr., Gandek B. Overview of the SF-36 health survey and the international quality of life assessment (IQOLA) project. J Clin Epidemiol. 1998;51(11):903–12. 9817107 10.1016/s0895-4356(98)00081-x

[38] Gunasekara FI, Richardson K, Carter K, Blakely T. Fixed effects analysis of repeated measures data. Int J Epidemiol. 2014;43(1):264–9. 24366487 10.1093/ije/dyt221

[39] Macedo M, Alves EDS, Jesus ITM, Inouye K, Brito TRP, Santos-Orlandi AAD. Frailty is associated with sociodemographic and health factors and related to the care context of older caregivers: a Brazilian cross-sectional study. Sao Paulo Med J. 2022;141(3):e202272. 36102461 10.1590/1516-3180.2022.0072.R1.06072022 PMC10065102

[40] Cameron AC, Trivedi PK. Microeconometrics using Stata. Texas: Stata Press College Station; 2009.

[41] Van Buuren S. Flexible imputation of missing data. CRC; 2018.

[42] Allison PD. Missing data. Sage; 2001.

[43] Brüderl J, Ludwig V. Fixed-effects panel regression. In: Best H, Wolf C, editors. The SAGE handbook of regression analysis and causal inference. London, England: SAGE Publications Ltd.; 2015. pp. 327–57.

[44] Pinquart M, Sorensen S. Spouses, adult children, and children-in-law as caregivers of older adults: a meta-analytic comparison. Psychol Aging. 2011;26(1):1–14. 21417538 10.1037/a0021863 PMC4449135

[45] Zwar L, Konig HH, Hajek A. Social support for informal caregivers of spouses and Parents(-in-Law) aged 60 years and older during the COVID-19 pandemic: findings from a representative German online survey. J Gerontol B Psychol Sci Soc Sci. 2023;78(2):264–79. 36074081 10.1093/geronb/gbac131 PMC9494367

[46] Yu DSF, Cheng ST, Wang J. Unravelling positive aspects of caregiving in dementia: an integrative review of research literature. Int J Nurs Stud. 2018;79:1–26. 29128685 10.1016/j.ijnurstu.2017.10.008

[47] Mehri N, Kinney J, Brown S, Rajabi Rostami M. Informal caregiving and All-Cause mortality: A Meta-Analysis of longitudinal Population-Based studies. J Appl Gerontol. 2021;40(7):781–91. 31838944 10.1177/0733464819893603

[48] Thornton J. WHO report shows that women outlive men worldwide. BMJ. 2019;365:l1631. 30952650 10.1136/bmj.l1631

[49] Bergmann M, Kneip T, De Luca G, Scherpenzeel A. Survey participation in the Survey of Health, Ageing and Retirement in Europe (SHARE), Wave 1–7. Based on Release 7.0.0. SHARE Working Paper Series: 41-2019. Munich: MEA, Max Planck Institute for Social Law and Social Policy.; 2019.

[50] Gangl M. Causal inference in sociological research. Ann Rev Sociol. 2010;36(1):21–47.

[51] Carbonneau H, Caron C, Desrosiers J. Development of a conceptual framework of positive aspects of caregiving in dementia. Dementia. 2010;9(3):327–53.

[52] Rowe JW, Kahn RL. Successful Aging1. Gerontologist. 1997;37(4):433–40. 9279031 10.1093/geront/37.4.433

[53] Ringer TJ, Hazzan AA, Kennedy CC, Karampatos S, Patterson C, Marr S, et al. Care recipients’ physical frailty is independently associated with subjective burden in informal caregivers in the community setting: a cross-sectional study. BMC Geriatr. 2016;16(1):186. 27855633 10.1186/s12877-016-0355-6 PMC5114771

[54] van Broese MI, de Boer A, Iedema J. Positive and negative evaluation of caregiving among three different types of informal care relationships. Eur J Ageing. 2013;10(4):301–11. 28804305 10.1007/s10433-013-0276-6 PMC5549208

[55] Tatangelo G, McCabe M, Macleod A, You E. I just don’t focus on my needs. The unmet health needs of partner and offspring caregivers of people with dementia: A qualitative study. Int J Nurs Stud. 2018;77:8–14. 28982034 10.1016/j.ijnurstu.2017.09.011

