# Supplementary material for: Seizing life with both hands: longitudinal analyses of grip strength among informal caregivers in Europe (SHARE)
Source: BMC Geriatr. 2025 Apr 30;25:298. doi: 10.1186/s12877-025-05949-y (PMC12042369; doi:10.1186/s12877-025-05949-y)
Supplement: Supplementary file 1 — Supplementary Material 1 [file 12877_2025_5949_MOESM1_ESM.docx]

**Supplementary Material**

**Table A1**

Missing values

|  | Complete sample | Caregivers inside the household^1^ | Caregivers outside the household |
| --- | --- | --- | --- |
|  | N=171,848 | N=10,148 | N=44,998 |
| Informal caregiving |  | 1245 (0.91) | 26,935 (15.67) |
| Who is cared for… |  | 821 (8.09) | 29 (0.06) |
| Age | 8 (0.00) | 1 (0.01) | 1 (0.00) |
| Gender | - | - | - |
| Education (ISCED 1997) | 2,319 (1.35) | 149 (1.47) | 335 (0.74) |
| BMI | 5,484 (3.20) | 366 (3.60) | 672 (1.50) |
| Marital status | 1,212 (0.7) | 66 (0.65) | 168 (0.38) |
| Current employment status | 2,388 (1.39) | 26 (0.26) | 134 (0.30) |
| Self-rated health | 578 (0.34) | 3 (0.03) | 6 (0.01) |
| Number of chronic diseases | 663 (0.39) | 11 (0.11) | 24 (0.05) |
| Hand grip strength | 13,998 (8.15) | 1,133 (11.16) | 1,379 (3.06) |
| Reason for missing grip strength measurement* |  |  |  |
| - Reason: refused to give measurement | 539 (3.85) | 32 (2.82) | 118 (8.56) |
| - Reason: unable to give measurement | 1,071 (7.65) | 73 (6.44) | 140 (10.15) |
| - Other reason given (e.g., felt it was not safe) | 9,273 (66.25) | 817 (72.11) | 912 (66.13) |
| - No reason given | 2879 (20.57) | 189 (16.68) | 153 (11.10) |

^1^ only those who were not living alone were asked if they were providing care inside the house; 10.28% of those having missing information on caregiving were living alone and therefore not asked if they provided care for anyone inside their household

*percentages refer to the group of participants with missing values in grip strength

Table A2

Main analysis of FE regression analysis for caregiving inside and outside the household stratified by three age groups (40 to 59, 60 to 79, 80 and older)

|  | Age group 1 (40 to 59) | | | Age group 2 (60 to 79) | | | Age group 3 (≥80) | | |
| --- | --- | --- | --- | --- | --- | --- | --- | --- | --- |
|  | (1) | | | (2) | | | (3) | | |
| VARIABLES | b | Robust SE | CI | b | Robust SE | CI | b | Robust SE | CI |
| Caregiving outside the household (ref. No) | 0.20* | (0.08) | 0.04 - 0.36 | 0.16** | (0.05) | 0.06 - 0.27 | 0.06 | (0.17) | -0.27 - 0.39 |
| Age at interview (in years) | -0.27*** | (0.01) | -0.30 - -0.24 | -0.42*** | (0.01) | -0.44 - -0.40 | -0.57*** | (0.02) | -0.62 - -0.52 |
| Constant | 50.07*** | (1.12) | 47.89 - 52.26 | 58.80*** | (0.77) | 57.30 - 60.31 | 70.51*** | (2.25) | 66.10 - 74.92 |
|  |  |  |  |  |  |  |  |  |  |
| Observations | 42,708 |  |  | 74,037 |  |  | 13,146 |  |  |
| N | 26,618 |  |  | 39,868 |  |  | 8,253 |  |  |
| R² | 0.0402 |  |  | 0.122 |  |  | 0.196 |  |  |
|  | Age group 1 (40 to 59) | | | Age group 2 (60 to 79) | | | Age group 3 (≥80) | | |
|  | (4) | | | (5) | | | (6) | | |
|  | b | Robust SE | CI | b | Robust SE | CI | b | Robust SE | CI |
| Caregiving inside the household (ref. No) | 0.15 | (0.15) | -0.14 - 0.44 | -0.00 | (0.11) | -0.22 - 0.21 | -0.17 | (0.26) | -0.68 - 0.34 |
| Age at interview (in years) | -0.28*** | (0.01) | -0.30 - -0.25 | -0.43*** | (0.01) | -0.45 - -0.41 | -0.68*** | (0.03) | -0.74 - -0.61 |
| Constant | 49.44*** | (1.03) | 47.42 - 51.46 | 59.59*** | (0.83) | 57.97 - 61.22 | 80.06*** | (3.29) | 73.62 - 86.50 |
|  |  |  |  |  |  |  |  |  |  |
| Observations | 44,231 |  |  | 69,374 |  |  | 8,199 |  |  |
| N | 25,839 |  |  | 35,007 |  |  | 5,295 |  |  |
| R² | 0.04 |  |  | 0.11 |  |  | 0.20 |  |  |

*Note*. Unstandardized regression coefficients of Fixed Effects regression analysis with robust standard errors in parentheses. All models are adjusted for age, marital status, current employment status, body mass index, activities requiring a moderate level of energy and sports or activities that are vigorous, and self-perceived health and number of chronic diseases. Level of significance: *** p<0.001, ** p<0.01, * p<0.05, + p<0.10.

Table A3

Asymmetric Fixed Effects regression analysis for informal caregiving outside the household

|  | (1) | | | (2) | | | (3) | | | (4) | | |
| --- | --- | --- | --- | --- | --- | --- | --- | --- | --- | --- | --- | --- |
|  | Main analysis | | | Moderator analysis | | | Main analysis | | | Moderator analysis | | |
| VARIABLES | b | Robust SE | CI | b | Robust SE | CI | b | Robust SE | CI | b | Robust SE | CI |
| Beginning caregiving outside the household (ref. No) | 0.34*** | (0.07) | 0.21 - 0.47 | 3.72*** | (0.78) | 2.20 - 5.24 |  |  |  |  |  |  |
| Ending caregiving outside the household (ref. No) |  |  |  |  |  |  | 0.13* | (0.06) | 0.00 - 0.25 | 4.20*** | (0.77) | 2.68 - 5.72 |
| Age at interview (in years) | -0.40*** | (0.01) | -0.41 - -0.39 | -0.53*** | (0.01) | -0.54 - -0.51 | -0.39*** | (0.01) | -0.40 - -0.38 | -0.52*** | (0.01) | -0.54 - -0.50 |
| Beginning caregiving outside the household (ref. No) x Age at interview (in years) |  |  |  | -0.05*** | (0.01) | -0.07 - -0.03 |  |  |  |  |  |  |
| Ending caregiving outside the household (ref. no) x age at interview (in years) |  |  |  |  |  |  |  |  |  | -0.06*** | (0.01) | -0.08 - -0.04 |
| Gender (ref. male) |  |  |  | - |  | - |  |  |  | - |  | - |
| Beginning caregiving outside the household (ref. No) x Gender (ref. Male) |  |  |  | -1.31 | (0.93) | -3.13 - 0.51 |  |  |  |  |  |  |
| Ending caregiving outside the household (ref. No) x Gender (ref. Male) |  |  |  |  |  |  |  |  |  | -2.61** | (0.91) | -4.39 - -0.83 |
| Gender (ref. Male) x Age at interview (in years) |  |  |  | 0.24*** | (0.01) | 0.22 - 0.26 |  |  |  | 0.24*** | (0.01) | 0.22 - 0.26 |
| Beginning caregiving outside the household (ref. No) x Gender (ref. Male) x Age at interview (in years) |  |  |  | 0.02 | (0.01) | -0.01 - 0.04 |  |  |  |  |  |  |
| Ending caregiving outside the household (ref. No) x Gender (ref. Male) x Age at interview (in years) |  |  |  |  |  |  |  |  |  | 0.04** | (0.01) | 0.01 - 0.06 |
| Constant | 56.71*** | (0.50) | 55.74 - 57.68 | 56.82*** | (0.50) | 55.85 - 57.79 | 56.30*** | (0.50) | 55.33 - 57.28 | 56.35*** | (0.50) | 55.38 - 57.32 |
| Observations | 152,105 |  |  | 152,105 |  |  | 152,037 |  |  | 152,037 |  |  |
| N | 68,144 |  |  | 68,144 |  |  | 68,145 |  |  | 68,145 |  |  |
| R² | 0.11 |  |  | 0.12 |  |  | 0.11 |  |  | 0.12 |  |  |

*Note*. Unstandardized regression coefficients of Asymmetric Fixed Effects regression analysis with robust standard errors in parentheses; only one-time transitions were included. All models are adjusted for age, marital status, current employment status, body mass index, activities requiring a moderate level of energy and sports or activities that are vigorous, and self-perceived health and number of chronic diseases. Level of significance: *** p<0.001, ** p<0.01, * p<0.05, + p<0.10.

Table A4

Asymmetric Fixed Effects regression analysis for informal caregiving inside the household

|  | (1) | | | (2) | | | (3) | | | (4) | | |
| --- | --- | --- | --- | --- | --- | --- | --- | --- | --- | --- | --- | --- |
|  | Main analysis | | | Moderator analysis | | | Main analysis | | | Moderator analysis | | |
| VARIABLES | b | Robust SE | CI | b | Robust SE | CI | b | Robust SE | CI | b | Robust SE | CI |
| Beginning caregiving inside the household (ref. No) | -0.05 | (0.11) | -0.27 - 0.16 | 5.94*** | (1.26) | 3.47 - 8.42 |  |  |  |  |  |  |
| Ending caregiving inside the household (ref. No) |  |  |  |  |  |  | -0.17 | (0.12) | -0.40 - 0.06 | 4.39*** | (1.29) | 1.86 - 6.92 |
| Age at interview (in years) | -0.39*** | (0.01) | -0.40 - -0.38 | -0.50*** | (0.01) | -0.52 - -0.48 | -0.39*** | (0.01) | -0.40 - -0.37 | -0.50*** | (0.01) | -0.52 - -0.48 |
| Beginning caregiving inside the household (ref. No) x Age at interview (in years) |  |  |  | -0.08*** | (0.02) | -0.12 - -0.05 |  |  |  |  |  |  |
| Ending caregiving inside the household (ref. no) x age at interview (in years) |  |  |  |  |  |  |  |  |  | -0.07*** | (0.02) | -0.10 - -0.03 |
| Gender (ref. male) |  |  |  | - |  | - |  |  |  | - |  | - |
| Beginning caregiving inside the household (ref. No) x Gender (ref. Male) |  |  |  | -4.44** | (1.54) | -7.46 - -1.43 |  |  |  |  |  |  |
| Ending caregiving inside the household (ref. No) x Gender (ref. Male) |  |  |  |  |  |  |  |  |  | -3.34* | (1.60) | -6.49 - -0.20 |
| Gender (ref. Male) x Age at interview (in years) |  |  |  | 0.23*** | (0.01) | 0.21 - 0.26 |  |  |  | 0.23*** | (0.01) | 0.21 - 0.25 |
| Beginning caregiving inside the household (ref. No) x Gender (ref. Male) x Age at interview (in years) |  |  |  | 0.06** | (0.02) | 0.02 - 0.10 |  |  |  |  |  |  |
| Ending caregiving inside the household (ref. No) x Gender (ref. Male) x Age at interview (in years) |  |  |  |  |  |  |  |  |  | 0.05* | (0.02) | 0.01 - 0.10 |
| Constant | 56.24*** | (0.57) | 55.12 - 57.37 | 56.38*** | (0.57) | 55.27 - 57.50 | 56.18*** | (0.57) | 55.05 - 57.30 | 56.34*** | (0.57) | 55.22 - 57.46 |
| Observations | 122,004 |  |  | 122,004 |  |  | 122,024 |  |  | 122,024 |  |  |
| N | 56,821 |  |  | 56,821 |  |  | 56,821 |  |  | 56,821 |  |  |
| R² | 0.10 |  |  | 0.11 |  |  | 0.10 |  |  | 0.11 |  |  |

*Note*. Unstandardized regression coefficients of Asymmetric Fixed Effects regression analysis with robust standard errors in parentheses; only one-time transitions were included. All models are adjusted for age, marital status, current employment status, body mass index, activities requiring a moderate level of energy and sports or activities that are vigorous, and self-perceived health and number of chronic diseases. Level of significance: *** p<0.001, ** p<0.01, * p<0.05, + p<0.10.

Table A5

Fixed Effects regression analysis with the complete sample (model 1), moderator analysis with age (model 2) and gender (model 3) and three-way interaction between caregiving, gender and age (mode 4) for the main predictor of *frequency of* *caregiving outside the household*

|  | (1) | | | (2) | | | (3) | | | (4) | | |
| --- | --- | --- | --- | --- | --- | --- | --- | --- | --- | --- | --- | --- |
|  | Max. of grip strength measure | | | Max. of grip strength measure | | | Max. of grip strength measure | | | Max. of grip strength measure | | |
| VARIABLES | b | Robust SE | CI | b | Robust SE | CI | b | Robust SE | CI | b | Robust SE | CI |
| Frequency of caregiving outside the household | -0.05 | (0.04) | -0.13 - 0.02 | 0.00 | (0.30) | -0.58 - 0.58 | -0.09 | (0.07) | -0.22 - 0.05 | -0.17 | (0.55) | -1.25 - 0.91 |
| Age at interview (in years) | -0.34*** | (0.01) | -0.37 - -0.32 | -0.34*** | (0.02) | -0.38 - -0.31 | -0.34*** | (0.01) | -0.37 - -0.32 | -0.46*** | (0.03) | -0.51 - -0.40 |
| Frequency of caregiving outside the household x Age |  |  |  | -0.00 | (0.00) | -0.01 - 0.01 |  |  |  | 0.00 | (0.01) | -0.02 - 0.02 |
| Gender (ref. male) |  |  |  |  |  |  | - |  | - | - |  | - |
| Frequency of caregiving outside the household x Gender (ref. male) |  |  |  |  |  |  | 0.06 | (0.08) | -0.10 - 0.22 | -0.11 | (0.64) | -1.35 - 1.14 |
| Gender (ref. male) |  |  |  |  |  |  |  |  |  | 0.19*** | (0.03) | 0.12 - 0.25 |
| Frequency of caregiving outside the household x Gender (ref. male) x Age |  |  |  |  |  |  |  |  |  | 0.00 | (0.01) | -0.02 - 0.02 |
| Constant | 54.58*** | (1.18) | 52.26 - 56.90 | 54.43*** | (1.42) | 51.66 - 57.20 | 54.59*** | (1.18) | 52.27 - 56.91 | 55.36*** | (1.42) | 52.57 - 58.15 |
| Observations | 42,735 |  |  | 42,735 |  |  | 42,735 |  |  | 42,735 |  |  |
| N | 28,614 |  |  | 28,614 |  |  | 28,614 |  |  | 28,614 |  |  |
| R² | 0.11 |  |  | 0.11 |  |  | 0.11 |  |  | 0.12 |  |  |

*Note*. Unstandardized regression coefficients of Fixed Effects regression analysis with robust standard errors in parentheses. Frequency of care provided outside the household measured as 1 daily, 2 weekly, 3 monthly, 4 less than monthly for at least one person supported by the caregiver. All models are adjusted for age, marital status, current employment status, body mass index, activities requiring a moderate level of energy and sports or activities that are vigorous, and self-perceived health and number of chronic diseases. Level of significance: *** p<0.001, ** p<0.01, * p<0.05, + p<0.10.

**Figure A1.**
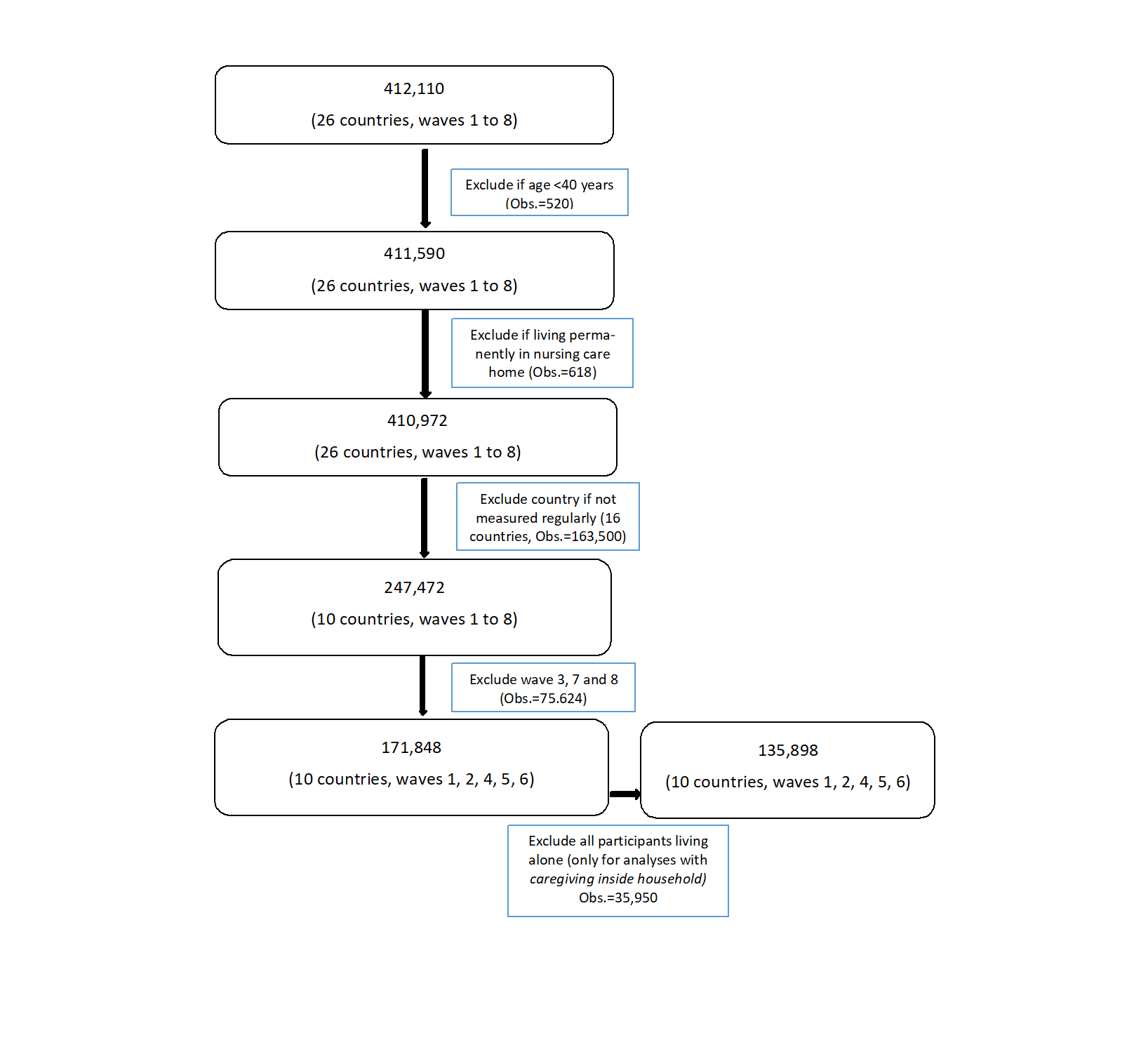
**Flow Chart for analytical sample**
